# A Cytofluorometric Study of Membrane Rafts in Human Monocyte Subsets in Atherosclerosis

**Published:** 2014

**Authors:** M. A. Chelombitko, V. S. Shishkina, O. P. Ilyinskaya, A. I. Kaminnyi, T. O. Pavlunina, N. N. Samovilova, E. V. Gracheva, E. M. Tararak, N. V. Prokazova

**Affiliations:** Biological Faculty, Lomonosov Moscow State University, Leninskie Gory 1, Bldg. 12, Moscow, 119991, Russia; Russian Cardiology Research and Production Complex, 3rd Cherepkovskaya Str., 15A, Moscow, 121552, Russia

**Keywords:** monocyte subsets, macrophages, membrane rafts, flow cytometry, atherosclerosis

## Abstract

The peripheral blood monocytes of atherosclerotic patients are pre-activated and have some of the features of tissue macrophages. Their adhesion to the endothelium is 1.5 times higher than that of monocytes from healthy subjects, and they express a number of receptors and antigens typical of tissue macrophages. Additionally, earlier we showed that the biosynthesis of gangliosides, whose main function is the formation of membrane rafts, is significantly activated in blood monocytes from atherosclerotic patients, as well as during the *in vitro *differentiation of normal monocytes into macrophages. In this study, we investigated the expression of membrane rafts on various monocyte subsets from healthy subjects and atherosclerotic patients. Based on flow cytometry results, the monocytes in the examined atherosclerotic patients were found to differ from those in healthy subjects by a twofold increase in the proportion of the intermediate subset (CD14^++^/CD16^+^) and by enhancement in the expression of the fractalkine receptor CX3CR1 on the intermediate and non-classical subsets (CD14^++^/CD16^+^ and CD14^+^/CD16^++^) (2.3 and 1.8 times, respectively). This suggests a pre-activated state of monocytes in atherosclerotic patients. At the same time, the expression of the membrane raft marker on the monocyte subsets was similar in both studied groups. However, a study of the *in vitro *differentiation of monocytes into macrophages showed that the membrane raft expression increased 2 times as early as on the 1st day of culturing and 3 times on the 7th day compared to that in freshly isolated monocytes. Therefore, it is suggested that monocytes in atherosclerosis accumulate gangliosides that are used to form membrane rafts during the macrophage differentiation after the migration of monocytes into the arterial intima.

## INTRODUCTION

Monocytes are immune system cells that play a key role in the formation of innate and adaptive immunity. Human blood monocytes are morphologically and functionally heterogeneous; several subsets can be distinguished based on the differential expression of CD14 (a component of the receptor complex that recognizes bacterial lipopolysaccharides) and CD16 (FcγRIII, the low affinity receptor for the Fc-fragment of IgG) [1, 2]. Recently, the Committee on Nomenclature of the International Union of Immunological Societies and the WHO adopted an official nomenclature, according to which monocytes are divided into three subsets: a classical (CD14^++^/CD16^-^ ), an intermediate (CD14^++^/CD16^+^), and a non-classical (CD14^+^/CD16^++^) (percentage of subsets is 83–85, 4–5, and 7–11%, respectively) [3, 4].

Clinical and experimental studies have shown that there is a significant increase in the number of monocytes in intermediate and non-classical subsets in infectious, autoimmune and inflammatory diseases, which may be an indication of the proinflammatory nature of their activity [5]. Furthermore, the monocytes of these populations are the main producers of proinflammatory cytokines: the tumor necrosis factor (TNF) and interleukin- 12 (IL-12) [6]. Monocytes play a critical role in the pathogenesis of atherosclerosis, because after they are attracted to the lipid and lipoprotein-enriched intimal areas of the arteries, they differentiate into macrophages under the influence of the macrophage colonystimulating factor (M-CSF) produced by the activated endothelium. Macrophages absorb oxidized lipoproteins and other lipids and form lipid saturated foam cells, which are the main cells of atherosclerotic plaques [7]. It is also obvious that the relative level of monocytes of minor subsets is significantly increased in atherosclerosis [8, 9]. In the peripheral blood of atherosclerotic patients, monocytes have been found to be preactivated and to exhibit some macrophage features. Their adhesion to the endothelium is 1.5 times higher than that of monocytes in healthy subjects. They express a number of receptors (Fcγ-receptor type I and II, ICAM) and an increased level of MHC II, which is typical of tissue macrophages [10-12; 12]. In addition, we demonstrated previously that the biosynthesis and level of gangliosides in the circulating monocytes of atherosclerotic patients are significantly higher than those in the monocytes of healthy subjects, similar to upon the *in vitro *differentiation of monocytes into macrophages [13, 14].

Gangliosides are sialic acid-containing glycosphingolipids that play an active role in the formation, stabilization, dynamics, and biological functions of membrane rafts. Due to the amide bond and bulk carbohydrate moiety in their molecule, gangliosides form in cell membranes, through a large number of hydrogen bonds, and conglomerate with cholesterol, sphingomyelin, and receptor proteins, the so-called membrane microdomains (rafts) that can move easily in the plane of the phospholipid membrane layer [15]. Therefore, raft gangliosides are involved in the processes of reception, adhesion, cell motility, and apoptosis. Their qualitative and quantitative composition changes dramatically during cell differentiation and transformation.

It is known that upon activation of lymphocytes, many receptors are transported into the rafts, where they acquire an active conformation and form complexes with other co-receptors and auxiliary proteins. Receptor phosphatidylinositol-anchored proteins, similarly to myristoylated and palmitoylated proteins, are permanent components of these membrane structures that are enriched in sphingolipids and cholesterol. Furthermore, transmembrane proteins are inductively involved in the rafts, thereby forming reception and adhesion foci [16].

In this paper, we conducted a comparative study of the expression of membrane rafts in different subsets of monocytes from the blood of atherosclerotic patients and healthy subjects, using flow cytometry, to elucidate the monocyte pre-activation mechanism in atherosclerosis.

## EXPERIMENTAL

**The study object**

In this study, peripheral blood samples were used that were obtained at the Russian Cardiology Research and Production Complex of the Russian Ministry of Health from patients with angiographically confirmed coronary atherosclerosis (n = 25) and apparently healthy donors (n = 15). In all cases, an informed patient consent was obtained. The mean age of the healthy subjects was 25 ± 3 years; the mean age of the patients was 55 ± 8 years. Since atherosclerosis is typical of almost all elderly people, the control group (healthy subjects) was composed of younger people.

**Isolation and flow cytometry analysis of human peripheral blood mononuclear leukocytes**

Mononuclear leukocytes were isolated from the peripheral blood by a traditional method in the Ficoll-Paque™ PLUS (Amersham Biosciences, USA) density gradient (1.077 g/L). The venous blood collected into tubes with 6% sodium EDTA (0.5 mL of EDTA per 10 mL of blood) was centrifuged at 400 g for 30 min. The blood cell pellet at the tube’s bottom was combined with phosphate buffered saline (PBS) to the original volume and carefully re-suspended. The resulting suspension was gently layered on Ficoll-Paque ™ PLUS at a 2.5 : 1 ratio and was centrifuged at 400 g for 30 min. The interphase layer containing the blood mononuclear cells was harvested and washed twice in PBS.

The expression of the surface markers of mononuclear leukocytes was analyzed by triple immunofluorescence staining. Membrane rafts were detected with the cholera toxin B subunit conjugated with Alexa Fluor 488 (Vybrant® Lipid Raft Labeling Kit, Molecular Probes, Inc.). Surface receptors were identified with specific, fluorescently labeled mouse monoclonal antibodies: CD14-PC5 (Beckman Coulter Inc.), CD16-PE (Beckman Coulter Inc.), CD16-FITC (Beckman Coulter Inc.), CCR5-PE (eBioscience Inc.), CX3CR1-PE (R & D Systems), and TLR-4-PE (eBioscience). After washing, the resultant precipitate was added with PBS (pH 7.2) containing 1% bovine serum albumin (BSA) in the amount of 100 μL of solution per sample. Cells were resuspended, and 100 μL aliquots of the suspension were transferred into 2 mL microtubes. Samples containing the cholera toxin B subunit (CTB) conjugated with Alexa Fluor 488 were incubated for 10 min; the remaining samples were incubated in the dark at 4 °C for 30 min. Further, each sample was added with 300 μL of 1% formalin, and the suspension was analyzed by flow cytometry. As the isotype control, mouse IgG isotype immunoglobulins labeled with dyes identical to labels of the monoclonal antibodies were used.

Flow cytometric studies were performed on a FACSCalibur flow laser cytometer (Becton Dickinson, USA) using identical instrument settings for all samples. The Summit V3.1 Built 844 (Cytomation Inc., USA) software was used for the analysis and visualization of the flow cytometry data. The monocyte region was identified based on the forward (FSC) and side (SSC) scatter parameters. The total monocyte population was identified based on CD14, in combination with SSC. Based on the expression level of CD14 and CD16 receptors, monocytes were divided into three subsets: CD14^++^/CD16^-^, CD14^++^/CD16^+^, and CD14^+^/CD16^++^. Separately, each subset was evaluated for the expression level of CCR2, TLR4, and CX3CR1 receptors using the mean fluorescence intensity (MFI).

**Culturing and immunocytochemical analysis of mononuclear cells in healthy subjects**

A portion of the isolated mononuclear cells from healthy subjects was plated on the bottom or glass of Petri dishes (o 60 mm) at a concentration of 2–2.5 million/ mL in a X-VIVO-10 culture medium (Lonza Biosciences, USA) containing 2 mM *L*-glutamine, 0.01 % fungizone, 1% penicillin/streptomycin mixture (Sigma reagents), and 10 ng/mL of M-CSF (Biosource) [13].

The dynamic pattern of co-expression of membrane rafts and various monocyte markers (CD14 and CD16) and the marker of differentiation of monocytes into macrophages (CD206) were analyzed at different stages of culturing (1^st^, 4^th^, and 7^th^ day) by triple immunofluorescence staining using specific, fluorescently labeled mouse monoclonal antibodies: CD14-PC5, CD16-PE, and CD206-PE (Beckman Coulter, USA). Rafts were identified using the cholera toxin B subunit conjugated with Alexa Fluor 488. For this, cells were detached from the plastic support by a rubber scriber and centrifuged at 400 g for 10 min. Then, the cells were stained as described above and analyzed on a FACSCalibur flow cytometer (Becton Dickinson).

The dynamic pattern of co-expression of membrane rafts with different markers of monocytes and macrophages at different stages of culturing (4^th^, 7^th^, and 12^th^ day) was analyzed by double immunofluorescence staining. For this, the cells plated on glass were stained using the cholera toxin B subunit conjugated with Alexa Fluor 488 and antibodies conjugated to phycoerythrin (PE) (to detect CD14-, CD16- and CD206-positive cells) and incubated at 37 0C in 5% CO_2_ for 30 min. Thereafter, the cells were washed three times with a warm medium and fixed in 10% formalin for 10 min. Then the samples were embedded in glycerin-gelatin and analyzed using a Leica DM 5000B fluorescence microscope (filters BP 450-490, LP 515 and BP 515-560, and LP 590) equipped with a DC 420 digital camera and an image analysis system. R-PE conjugated IgGs of the same subclass as the specific antibodies were used as the isotype control.

**Statistical analysis**

The statistical analysis of the data was performed using the Excel and Statistica 7.0 software. The two-sided Mann-Whitney U-test was used to estimate the statistical significance of intergroup differences. The differences were considered statistically significant at P < 0.05. The data are presented as the arithmetic mean and its standard deviation (M ± SD).

## RESULTS AND DISCUSSION

**Flow cytometry analysis of the monocyte subsets of patients and healthy subjects**

**Fig. 1:**
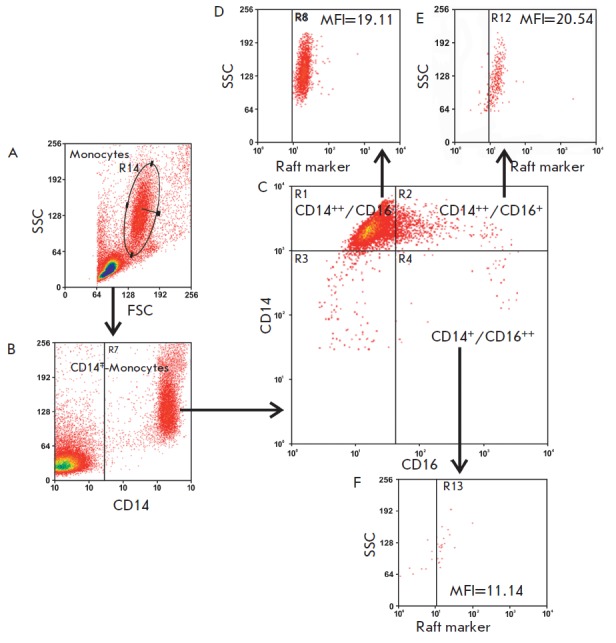
Multistep gating of monocyte subsets. (A) Identification of the monocyte region based on the forward scatter (FSC) and side scatter (SSC) parameters; (B) Gating of the total population based on CD14 positive events; (C) Identification of monocyte subsets by the expression level of the surface markers CD14 and CD16; (D–F) Gating in the analysis of the expression of various receptors and membrane rafts on monocyte subsets based on the SSC parameters and positive events (illustrated by an example of the GM1 raft marker)

The monocytes and their subsets were identified by flow cytometry using the forward and side scatter parameters and the expression level of the surface markers CD14 and CD16. A gating strategy used to identify the classical (CD14^++^/CD16^-^), intermediate (CD14^++^/CD16^+^), and non-classical (CD14^+^/CD16^++^) monocyte subsets is presented in *Figs. 1A–C*. The obtained cytofluorograms and percentage ratio of the subsets are consistent with published data [3, 4].

*Figures 1D–F *present the results of the flow cytometry analysis of the membrane rafts on monocyte subsets using monoclonal antibodies to CD14 and CD16 and the cholera toxin B subunit, which identifies membrane rafts. To detect rafts on the membranes of whole cells, a commonly used method based on the very high affinity (10^-12^ M) of the cholera toxin B subunit to the GM1 ganglioside was utilized. The method is based on their interactions on the cell membrane, although the level of this ganglioside compared to that of other gangliosides in the plasma membrane of blood cells is low [17]. Therefore, the use of GM1 as a raft marker is related not so much to its significance for the raft structure as to its availability as a reactant [18]. The analysis of the results by the Summit V3.1 Built 844 software demonstrated that all of the monocyte subsets expressed GM1, a marker of membrane rafts (*Figs. 1D–F*).

**Fig. 2:**
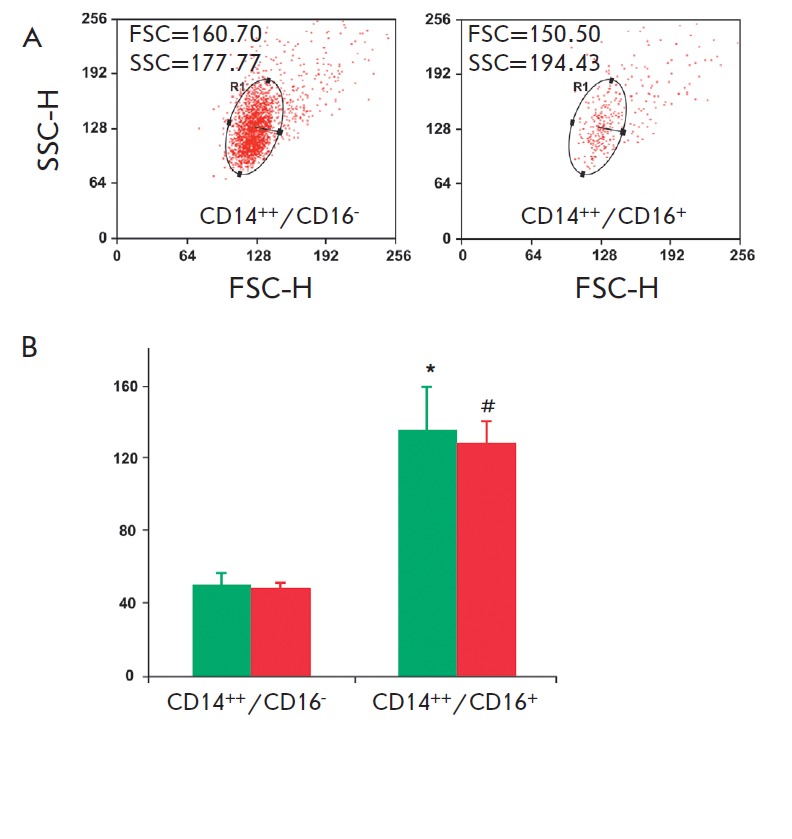
Blood cell subsets from a healthy subject located outside a monocyte gate. (A) Characteristics of CD14^++^/ CD16– and CD14^++^/CD16^+^ cell subsets based on their granularity (SSC) and size (FSC); (B) Cumulative data on the fluorescence intensity of the raft marker (GM1) on CD14^++^/CD16– and CD14^++^/CD16^+^ cells in 15 healthy subjects (green bars) and 25 atherosclerotic patients (red bars). Values are presented as M ± SD. *,#P < 0.5

Interestingly, our data are partly inconsistent with the results obtained previously by Moreno-Altamirano* et al. *[19], who divided the monocytes of healthy subjects into two populations based on the expression of rafts: CD14^+^/GM1^+^ (95.5% with a low raft expression) and CD14^+^/GM1^++^ (2.5% with a high raft expression). To analyze the rafts, they, like us, used a fluorescently labeled cholera toxin B subunit. We observed a different pattern (*Figs. 1D–F*): the classical and intermediate monocyte subsets had the same raft expression; the low expression was typical of the non-classical subset only. This contradiction may be due to the different gating strategy used by Moreno-Altamirano *et al*. Indeed, as seen from the data in *Figs. 2A *and *B*, based on the granularity (SSC) and size (FSC) parameters, which were different from those of the monocytes in the gate, we identified the so-called non-gate classical (CD14^++^/CD16^-^) and intermediate (CD14^++^/CD16^+^) monocyte subsets. In this case, the latter subset had a higher expression of the marker rafts than the classical one. It is most likely that Moreno-Altamirano *et al*. [19], contrary to us, probably took into account the cells of non-gated subsets. We observed the monocytes of nongated subsets in equal amounts both in patients and in healthy subjects (*Fig. 2B*). The properties and functions of this population need to be further studied.

**Fig. 3:**
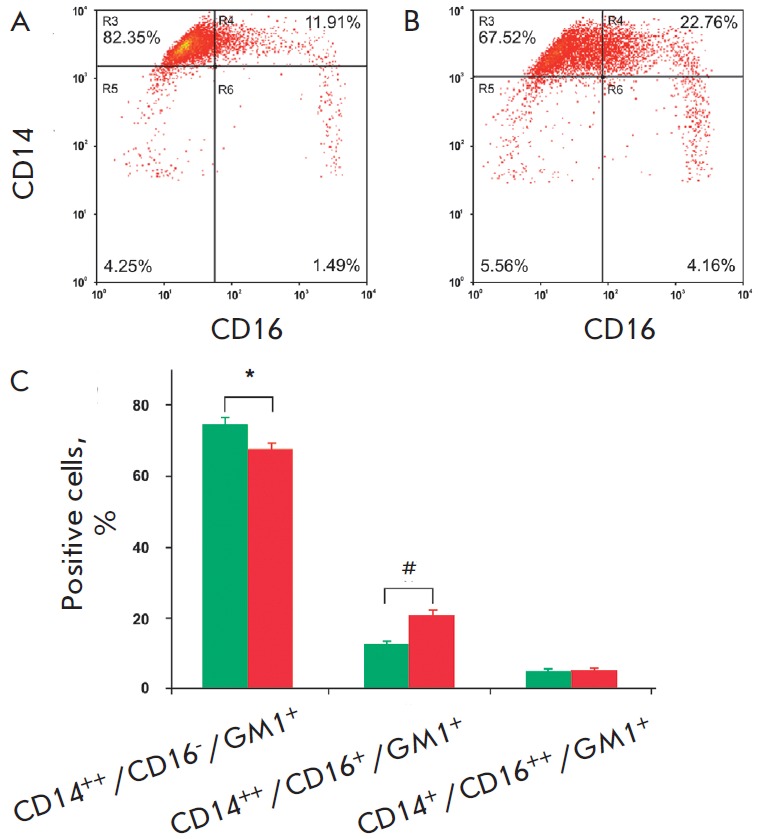
Monocyte subsets from healthy subjects and atherosclerotic patients. Typical cytofluorograms of monocyte subsets from a healthy subject (A) and an atherosclerotic patient (B); (C) Cumulative data on the percentage ratio of monocyte subsets in 15 healthy subjects (green bars) and 25 atherosclerotic patients (red bars). Values are presented as M ± SD. *,#P < 0.05

*Figures 3A *and *B *provide the typical cytofluorograms of the monocyte subsets of a healthy subject and an atherosclerotic patient. The data in *Fig. 3B* demonstrate that the percentage of intermediate subset monocytes is significantly increased (20.7 ± 7.0%) and the percentage of classical subset monocytes is decreased (68.6 ± 7.9%) in patients with atherosclerosis compared to those of healthy subjects (12.8 ± 3.4 and 74.8 ± 7.6%, respectively). Therefore, atherosclerosis is apparently accompanied by a redistribution of monocyte subsets: the proportion of monocytes with an intermediate phenotype (CD14^++^/CD16^+^) is increased due to the reduction in the proportion of the main subset of classical monocytes (CD14^++^/CD16^-^). The level of non-classical subset monocytes was identical in patients and healthy subjects.

These findings are consistent with the results of a series of studies that explored monocyte subsets in atherosclerosis. For example, the high level of intermediate subset monocytes (CD14^++^/CD16^+^) was found to be associated with an increase in the body mass index and thickening of the intima-media complex. Clinical data indicate a higher level of CD14^++^/CD16^+^ monocytes and the total population of CD16^+^ monocytes in patients with coronary heart disease compared to healthy subjects. An increase in the number of monocytes of the CD16^+^ subsets was associated with the predominance of unstable plaques in the coronary arteries and an unfavorable prognosis in the coronary heart disease [3, 6, 20]. It should also be noted that, until recently, the CD14^++^/CD16^+^ and CD14^+^/CD16^++^ monocyte subsets were analyzed together in many clinical studies, because a standard gating protocol was not adopted in the analysis of flow cytometry data to identify monocyte subsets. This did not allow researchers to draw an unambiguous conclusion on the role of individual CD16+ subsets in the pathogenesis of atherosclerosis. As seen from *Fig. 3B*, no significant differences in the percentage of non-classical subset monocytes in healthy subjects and in atherosclerotic patients were found.

**Expression of cytokine receptors on monocyte subsets**

The function of chemokines and their receptors is to attract specifically different monocyte subsets to the inflammatory area [21]. Monocyte populations are known to differ in their expression of chemokine receptors [22]. For example, the classical CD14^++^/CD16^-^ subset is characterized by a high level of CCR2, the receptor of the monocyte chemoattractant protein-1 (MCP-1), a moderate expression of CX3CR1, the fractalkine receptor, and a low level of CCR5, the receptor of the inflammatory cytokines CCL3, CCL4, CCL8, and CCL3. The CD16+ subsets are CCR 2 negative and express a high level of the CX3CR1 and CCR5 receptors. These two receptors were also found to play a prominent role in the formation of atherosclerotic lesions, because their ligands are found in the atherosclerotic plaques and are expressed by endothelial cells after their activation by cytokines [23, 24]. In connection with this, the expressions of CCR5, CX3CR1, and the LPS receptor (TLR4), which is common to all monocytes, were studied on the monocytes of healthy subjects and arteriosclerotic patients using flow cytometry with triple staining by monoclonal antibodies. No significant differences in the CCR5 and TLR4 expression by the monocytes of atherosclerotic patients and healthy subjects were found among all the subsets (data not shown).

**Fig. 4:**
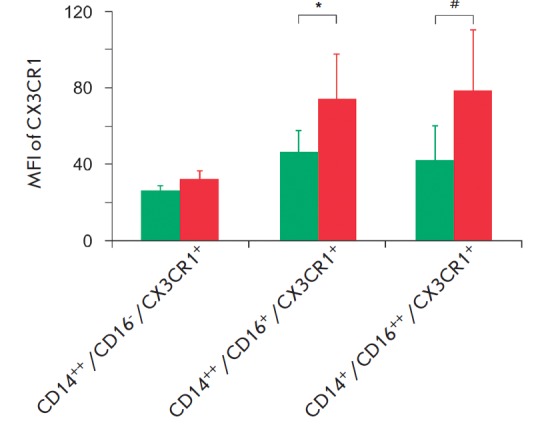
Fractalkine receptor (CX3CR1) expression on monocyte subsets in 15 healthy subjects and 25 atherosclerotic patients. The differences are statistically significant for the CD16^+^ cells of healthy subjects (green bars) and atherosclerotic patients (red bars). Values are presented as M ± SD. P < 0.05

The expression of CX3CR1 (fractalkine receptor) on the intermediate and non-classical subsets was higher than that on classical monocytes, which is consistent with the concept of its localization on CD16^+^ monocytes [23]. In this case, both CD16^+^ monocyte subsets in pa tients had a CX3CR1 fluorescence intensity 2 times higher than that of the monocytes in healthy subjects (*Fig. 4*).

An unambiguous role of CX3CR1/CX3CL1 in the formation of atherosclerotic lesions in human vessels was also demonstrated in experimental atherosclerosis in mice [24]. Therefore, our findings confirm the results of previous studies.

**Expression of lipid rafts on monocyte subsets**

Previously, we found that the biosynthesis of gangliosides whose main function is the formation of lipid rafts is significantly activated in the blood monocytes of patients with atherosclerosis [15]. On this basis, we suggested that pre-activation of circulating monocytes in atherosclerosis is accompanied by an increase in the number of the lipid rafts necessary for the functioning of membrane proteins.

Many antigens and receptors of monocytes, such as CD14, CD32, CD64, CD11/CD18, and the major histocompatibility complex class II (MHC II), were previously demonstrated to be permanent components of membrane rafts [25, 26]. When monocytes are activated, additional protein molecules are transported to the plasma membrane. For example, under certain conditions, CD16 are mobilized from cytosol depots to membrane rafts [27]. Activation and functioning of monocytes require the integration of individual rafts into large platforms with the involvement of additional protein components [28, 29]. Disruption of rafts through treatment of cells with nystatin or methyl cyclodextrin (cholesterol binding compounds), conversely, results in a loss of the association of receptors in lipid platforms and, consequently, in a disruption of signal transduction and cellular responses to specific ligands [10].

As seen from the data shown in *Fig. 3B*, the percentage ratio among the subsets of monocytes expressing GM1^+^ rafts (CD14^++^/CD16^-^/GM1^+^, CD14^++^/CD16^+^/ GM1^+^, and CD14^+^/CD16^++^/GM1^+^) in healthy subjects and in atherosclerotic patients did not differ; however, a redistribution of monocytes among the subsets of CD14^++^/CD16^-^, CD14^++^/CD16^+^ and CD14+/CD16^++^ occurred in atherosclerosis (see above).

**Fig. 5:**
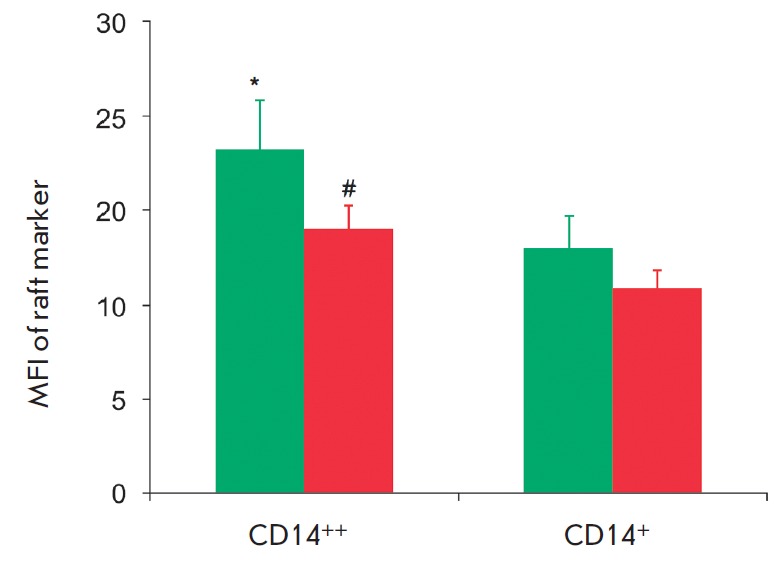
Cumulative data on the mean fluorescence intensity (MFI) of the raft marker (GM1) on monocyte subsets in 15 healthy subjects (green bars) and 25 atherosclerotic patients (red bars). Values are presented as M ± SD. *,#P < 0.05

The analysis of fluorescence after triple staining with antibodies to CD14 and CD16 and the cholera toxin B subunit demonstrated that the mean fluorescence intensity (MFI) of rafts on monocytes with a high expression of CD14 was higher than that on monocytes with a low expression of CD14 both in patients and in healthy subjects (1.4 and 1.27 times, respectively) (*Fig. 5*). Based on these data, it may be concluded that the direct relationship between the accumulation of gangliosides in monocytes in atherosclerosis and an increase in the number of rafts in the membranes of these cells is not obvious. We assumed that this ganglioside pool may be realized in the form of lipid rafts as a result of the differentiation of pre-activated monocytes in the arterial intima during the atherogenesis.

**Flow cytometry analysis of the membrane rafts of cultured monocytes/macrophages in healthy subjects**

Previously, we observed a significant increase in the synthesis of gangliosides during the *in vitro *differentiation of human monocytes into macrophages [14]. We also found that the mRNA level of GM3 synthase (a key enzyme of the ganglioside synthesis) was significantly higher in cultured monocytes/macrophages than in freshly isolated monocytes and in the intima of an atherosclerotic plaque compared to the intima of unaffected regions of the human aorta [30]. It should be noted that macrophages are the main cells in the intima of the atherosclerotic arteries, whereas they are actually absent in the normal intima.

**Fig. 6:**
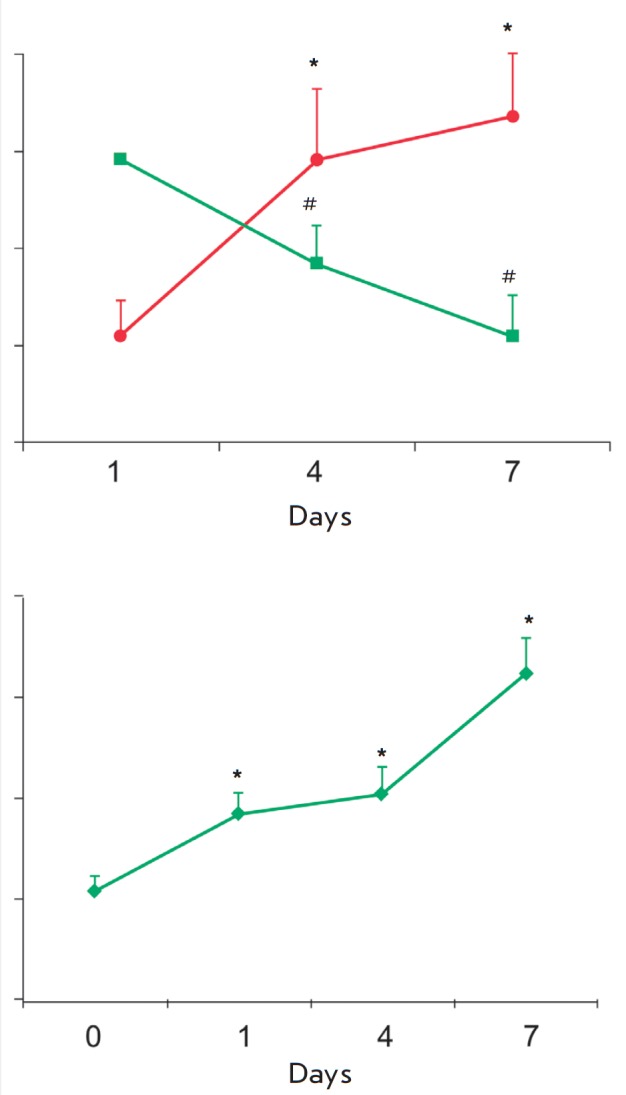
Cytofluorometric analysis of the *in vitro *differentiation of cultured monocytes from healthy subjects. (A) Change in the proportion of CD14^+^/CD16^+^ (sqare) and CD14^+^/CD206^+^ cells (circle) during the differentiation (n=3). *,#The differences are statistically significant between the 1st and the 4th day and the 1^st^ and 7^th^ day; (B) Change in the raft marker expression on CD14^+^/CD206^+^ cells during the differentiation (n=3). *The difference is significant between the reference point and 1st day, and the 1^st^ and 7^th^ day. Values are presented as M ± SD. *,#P < 0.05

This study demonstrated, using the flow cytometry analysis of cultured monocytes of healthy subjects, a reduction in the proportion of CD14+/CD16+ cells and an increase in the proportion of CD14^+^/CD206^+^ cells (*Fig. 6A*), which indicates a differentiation of monocytes into macrophages [30]. Increasing MFI of rafts by 2 times as early as after 24 h and by 3 times on the 7th day of culturing compared to freshly isolated monocytes (*Fig. 6B*) was revealed by triple staining with antibodies to CD14 and CD206 (macrophage differentiation marker) and the fluorescently labeled cholera toxin B subunit.

These findings, in conjunction with our earlier results [13, 14, 30], indicate a probable relationship between biosynthesis activation and, as a consequence, an increase in the level of gangliosides and a higher expression of lipid rafts in cultured macrophages compared to freshly isolated monocytes. An increasing amount of data confirms that monocytes are already pre-activated, circulating before their migration to inflammation areas during inflammatory pathologies accompanied by an increased production of cytokines, such as TNF-α, M-CSF, or IL-6 [10-12]. In this regard, it seems likely that the previously found accumulation of gangliosides in the activated monocytes of atherosclerotic patients [13] is necessary for the formation of membrane rafts after the infiltration of monocytes into the vascular wall intima with their subsequent differentiation into macrophages.

In previous studies, we found, using methods of immunocytochemistry with specific antibodies to the main ganglioside of monocytes/macrophages, that the intima of human atherosclerotic lesions contains a large number of cells with a high expression of this ganglioside. The study of the phenotype of these cells demonstrated that the majority of them were macrophages [31,32].

**Fig. 7:**
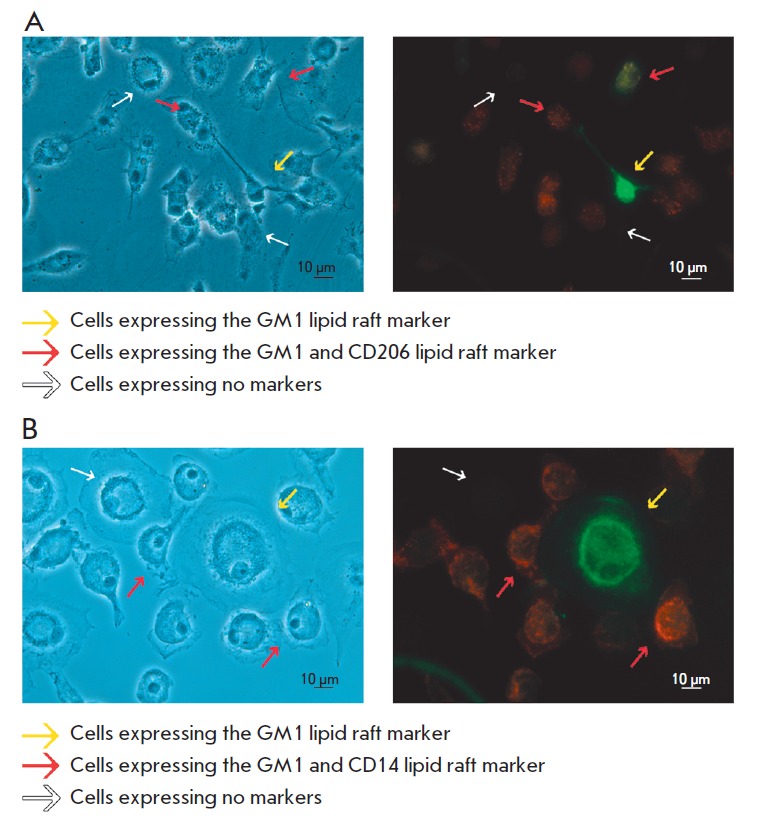
Double immunofluorescence staining of cultured monocytes/macrophages from a healthy subject. Double staining with the cholera toxin B-subunit, which reveals GM1ganglioside, and monoclonal antibodies, which recognize the differentiation marker CD206 (A) or the monocyte/macrophage marker CD14 (B). The left images are obtained by phase contrast microscopy of the same cells. The length of the bar is 10 μm

A study of the expression of differentiation and lipid raft markers using double staining of monocytes/macrophages revealed that a significant portion of CD14^+^/ SD206^+^ cells was raft-positive (*Figs. 7A *and *B*). A small number of cells of different morphologies with a high expression of the raft marker were present at all stages of culturing. Among these cells, cells expressing and non-expressing markers of macrophages were present.

## CONCLUSIONS

The flow cytometry study based on a single strategy of gating monocyte subsets demonstrated that three monocyte subsets (classical, intermediate, and non-classical) can be identified in the peripheral blood of both atherosclerotic patients and healthy subjects. In patients with atherosclerosis, an increase in the proportion of the intermediate subset (CD14^++^/CD16^+^) and a decline in the proportion of the classical subset (CD14^++^/CD16^-^) of monocytes were observed compared to healthy subjects. In them, the monocytes of the intermediate subset were characterized by a higher expression of the fractalkine receptor CX3CR1 compared to healthy subjects. The monocytes of patients and healthy subjects did not differ in the expression of membrane rafts. However, CD14^++^ monocytes differed from CD14^+^ monocytes by a higher expression of rafts. In addition, the so-called non-gate classical (CD14^++^/ CD16^-^) and intermediate (CD14^++^/CD16^+^) monocyte subsets were identified in both of the studied groups, with the latter subset having a higher expression of the raft marker. During culturing of monocytes/macrophages in the presence of M-CSF, their activation with further differentiation accompanied by a threefold increase in the expression of membrane rafts occurred. In conjunction with our previous data, it may be concluded that the accumulation of gangliosides, which are essential components of rafts, in pre-activated monocytes of atherosclerotic patients did not result in an increase in the expression of membrane rafts. However, the data obtained in this work suggest that during the differentiation of monocytes into macrophages, an accumulated pool of gangliosides is realized in macrophages in the form of the rafts required for activating the adhesion and phagocytosis.
